# Supplementary material for: Using set theory to reduce redundancy in pathway sets
Source: BMC Bioinformatics. 2018 Oct 19;19:386. doi: 10.1186/s12859-018-2355-3 (PMC6194563; doi:10.1186/s12859-018-2355-3)
Supplement: Supplementary file 1 — Table S1. Enriched pathways from the osteoarthritis dataset (p-value< 0.05). The set cover column indicated the 23 pathways that were included in the set cover. (DOCX 19 kb) [file 12859_2018_2355_MOESM1_ESM.docx]

Table S1: Enriched pathways from the osteoarthritis dataset (p-value<0.05). The set cover column indicated the 23 pathways that were included in the set cover.

| p-value | Set cover | pathway |
| --- | --- | --- |
| **0.0000** | **1** | **extracellular matrix organization** |
| 0.0000 | 0 | collagen biosynthesis and modifying enzymes |
| 0.0000 | 0 | collagen formation |
| **0.0000** | **1** | **gpcr signaling g alpha q** |
| **0.0000** | **1** | **signal transduction** |
| **0.0000** | **1** | **protein digestion and absorption homo sapiens human** |
| **0.0000** | **1** | **pathways in cancer homo sapiens human** |
| 0.0000 | 0 | gpcr signaling cholera toxin |
| 0.0000 | 0 | gpcr signaling pertussis toxin |
| 0.0000 | 0 | class b 2 secretin family receptors |
| 0.0000 | 0 | gpcr ligand binding |
| 0.0001 | 0 | gpcr signaling g alpha s epac and erk |
| 0.0001 | 0 | gpcr signaling g alpha s pka and erk |
| 0.0003 | 0 | integrin cell surface interactions |
| **0.0003** | **1** | **vitamin d receptor pathway** |
| 0.0004 | 0 | signaling by gpcr |
| **0.0006** | **1** | **integrin** |
| 0.0006 | 0 | basal cell carcinoma homo sapiens human |
| 0.0010 | 0 | ecm proteoglycans |
| **0.0015** | **1** | **wnt signaling network** |
| **0.0016** | **1** | **o linked glycosylation** |
| **0.0022** | **1** | **ecm receptor interaction homo sapiens human** |
| **0.0027** | **1** | **small cell lung cancer homo sapiens human** |
| **0.0067** | **1** | **wnt signaling pathway** |
| 0.0082 | 0 | degradation of the extracellular matrix |
| **0.0115** | **1** | **signaling pathways regulating pluripotency of stem cells homo sapiens** |
| **0.0136** | **1** | **beta1 integrin cell surface interactions** |
| **0.0142** | **1** | **complement and coagulation cascades homo sapiens human** |
| **0.0146** | **1** | **cell adhesion molecules cams homo sapiens human** |
| **0.0262** | **1** | **pi3k akt signaling pathway homo sapiens human** |
| 0.0354 | 0 | collagen degradation |
| 0.0381 | 0 | wnt5a dependent internalization of fzd2 fzd5 and ror2 |
| **0.0389** | **1** | **hippo signaling pathway homo sapiens human** |
| 0.0415 | 0 | gpcr downstream signaling |
| **0.0415** | **1** | **benzo a pyrene metabolism** |
| 0.0430 | 0 | o linked glycosylation of mucins |
| **0.0430** | **1** | **axon guidance** |
| **0.0430** | **1** | **prostaglandin synthesis and regulation** |
| 0.0436 | 0 | activation of trka receptors |
| **0.0436** | **1** | **neuroactive ligand receptor interaction homo sapiens human** |
| 0.0445 | 0 | small ligand gpcrs |
| **0.0453** | **1** | **wnt signaling pathway and pluripotency** |
